# Reproductive Performance of Triplet-Bearing Ewes on Commercial Farms and Research Priorities Identified by Sheep Producers to Improve the Survival of Triplet-Bearing Ewes and Their Lambs

**DOI:** 10.3390/ani13071258

**Published:** 2023-04-05

**Authors:** Andrew N. Thompson, Travis Allington, Sarah Blumer, Jo Cameron, Gavin Kearney, Lyndon Kubeil, Amy Lockwood, Jason Trompf, Emma Winslow, Paul Kenyon

**Affiliations:** 1 Centre for Animal Production and Health, Murdoch University, Murdoch, WA 6150, Australia; travis.allington@murdoch.edu.au (T.A.); sarah.blumer@murdoch.edu.au (S.B.); a.lockwood@murdoch.edu.au (A.L.); 2 Department of Economic Development, Jobs, Transport and Resources, 915 Mount Napier Road, Hamilton, VIC 3300, Australia; jo.cameron@ecodev.vic.gov.au; 3 Independent Researcher, 36 Payne Road, Hamilton, VIC 3300, Australia; gke29755@bigpond.net.au; 4 Department of Economic Development, Jobs, Transport and Resources, Sydney Rd, Benalla, VIC 3672, Australia; lyndon.kubeil@agriculture.vic.gov.au; 5 JT Agri-Source, Bradley Drive, Mill Park, VIC 3082, Australia; j.trompf@latrobe.edu.au; 6 South Australian Research and Development Institute, Naracoorte, SA 5271, Australia; emma.winslow@sa.gov.au; 7 School of Agriculture and Environment, Massey University, Palmerston North 4472, New Zealand; p.r.kenyon@massey.ac.nz

**Keywords:** triplet-bearing ewes, ewe mortality, lamb survival, producer consultation, needs analysis, research priorities

## Abstract

**Simple Summary:**

The proportion of ewes conceiving triplets is increasing on farms across Australia as sheep producers adopt more fecund genetics and ewe management practices to increase lamb output per ewe mated. The aims of this research were to consult producers to quantify rates of ewe and lamb mortality in this cohort and identify priorities for future research to reduce these losses. Surveys of producers with experience in managing triplet-bearing ewes indicated the average mortality of triplet-bearing ewes was 6.4%, and the survival of triplet-born lambs was 59%. There was significant variation in the actual targets adopted by different producers for ewe condition score at lambing, mob size during lambing and feed-on-offer at lambing, and no differences in the average rate of mortality of triplet-bearing ewes or lamb survival between producers that prioritised adoption of certain management practices. The highest priorities for further research identified by producers from surveys, workshops and a webinar were ewe condition score, mob size, feed-on-offer at lambing and mineral supplementation.

**Abstract:**

Consultation with sheep producers was used to quantify the mortality of triplet-bearing ewes and their lambs, identify management practices adopted by producers to reduce these losses and prioritise future research needs to improve the survival of triplet-bearing ewes and their lambs. Surveys were completed by 64 producers across Australia who identified and separated triplet-bearing ewes from twin-bearing ewes in 2017 and/or 2018. On average, 5.9% of all ewes mated were identified as carrying triplets (6.6% of non-Merino ewes and 2.9% of Merino ewes). The average mortality of triplet-bearing ewes was 6.4%, and ewe mortality did not differ significantly between ewe breeds. The average survival of triplet-born lambs was 59%, and survival was significantly higher for lambs from non-Merino compared to Merino ewes (60.1 vs. 52.9%, *p* < 0.05). The key strategies adopted to reduce the mortality of triplet-bearing ewes and their lambs included management of condition score, feed-on-offer, mob size at lambing and use of shelter. There were no differences (*p* > 0.05) in the average mortality of triplet-bearing ewes or their lambs between producers that prioritised the adoption of certain management practices. However, significant variation existed between producers in their targets at lambing for ewe condition score (2.8 to 3.5), mob size (10 to 150 ewes) and feed-on-offer (800 to 2500 kg dry matter/ha). Overwhelmingly, the highest priorities for further research identified by producers from surveys, workshops and a webinar were ewe condition score, mob size, feed-on-offer at lambing and mineral supplementation. This study informs benchmarks for mortality of triplet-bearing ewes and their lambs under extensive grazing conditions in Australia, and the priorities for future research to reduce these losses.

## 1. Introduction

Increasing lamb production can improve the profitability of sheep enterprises, especially for non-Merino sheep and when sheep meat prices are high [1,2]. Lamb marking rates in Australia have increased by more than 10% over the last 15 years [3]. This has resulted from the widespread adoption of practices to improve ewe nutrition before joining and during pregnancy [4,5], improved management during lambing [6,7,8] and increased use of sires with higher breeding values for the number of lambs born and weaned. In addition, there has been significant displacement of Merino sheep by more fecund maternal ewe types [9]. These increases in fecundity are associated with an increase in the proportion of multiple-bearing ewes, including those carrying triplets [10], which can result in higher rates of mortality of both ewes and lambs [11]. High rates of mortality of triplet-bearing ewes and their lambs limit the potential productivity gains and is an animal welfare issue. 

Triplet lambs are born lighter, are more metabolically challenged, have lower body temperature and impaired behavioural development, and receive less colostrum and milk than their twin counterparts. Combined, these result in higher rates of mortality of triplet-born lambs [11]. Kenyon et al. [11] reported that the average birthweight of triplet-born lambs was 66% and 81% that of the birthweight of single- and twin-born lambs, and the average mortality rates of single-, twin- and triplet-born lambs were 10%, 15% and 33%, respectively. Triplet-bearing ewes are under greater nutritional stress in late pregnancy than twin-bearing ewes, as their increased nutritional demand is generally not matched by an increase in nutrient intake [11]. This nutritional stress also contributes to higher mortality of triplet-bearing ewes compared to single- and twin-bearing ewes [12]. There is, however, a lack of robust data on the magnitude and cause of mortality for triplet-bearing ewes and their lambs, particularly in Australia.

Management guidelines based on achieving ewe condition score, feed-on-offer (FOO) or mob size targets at lambing have been developed for single- and twin-bearing Merino and non-Merino ewes in Australia [1,13,14]. However, targets for triplet-bearing ewes are unknown. To develop these targets, it is likely that further research is needed to examine the impacts of ewe condition score, FOO and mob size at lambing on the survival of the triplet-bearing ewe and her lambs [11]. In addition, more knowledge of the potential impacts of shelter, other paddock characteristics and human intervention are required. There appears to have been no attempt to date to survey the management practices for triplet-bearing ewes that have already been adopted by sheep producers in Australia, nor a formal research-needs analysis based upon direct producer input. This study aimed to (i) identify current practices adopted for the management of triplet-bearing ewes; (ii) quantify the mortality of triplet-bearing ewes and their lambs on commercial farms in Australia; and (iii) identify research priorities to improve the survival of triplet-bearing ewes and their lambs from producer consultation. 

## 2. Materials and Methods

### 2.1. Benchmark Surveys

Telephone or in-person surveys were conducted in 2018 for 95 producers who had either pregnancy scanned to identify some triplet-bearing ewes and managed them separately from twin-bearing ewes (‘Separated’ management; *n* = 64) or who always scanned for multiple-bearing ewes only and did not manage triplet-bearing ewes separately from twin-bearing ewes (‘Combined’ management; *n* = 31). Contact details for producers to survey were obtained mostly through professional networks. Pregnancy scanning contractors and sheep production consultants also provided contacts of their clients who had confirmed their interest and eligibility for participation in the surveys. The ewe management system for a farm was classified as ‘Separated’ if the triplet-bearing ewes from at least one ewe flock on their farm were identified and separated from twin-bearing ewes in either 2017 or 2018, where ‘flock’ represented all adult Merino or non-Merino ewes on a farm. In other words, over the two years, a farm classified as ‘Separated’ could include both separated and combined flocks. 

**Figure 1:**
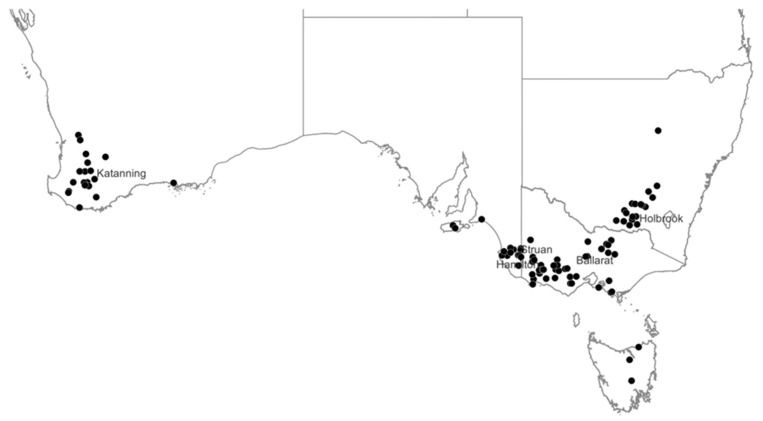
Location of participants of benchmarking surveys (black circle; *n* = 95) and workshops (*n* = 5) across southern Australia.

Survey participants were from New South Wales (NSW, *n* = 17), South Australia (SA, *n* = 15), Tasmania (TAS, *n* = 4), Victoria (VIC, *n* = 40) and Western Australia (WA, *n* = 19) (Figure 1). The producers surveyed included 26 farms with Merino ewes, 50 farms with non-Merino ewes and 19 farms with both Merino and non-Merino ewes. These surveys collected background farm-level information including location, total farm area, percentage of farm cropped, ewe breed, number of ewes mated, and date and length of the mating period for both the 2017 and 2018 breeding seasons. Data collected included reproductive rate (number of foetuses conceived per 100 ewes mated) and lamb marking percentage (number of lambs marked per 100 ewes mated) for each flock on the farm, and ewe mortality and lamb survival for single-, twin- and triplet-bearing ewes, where possible. The producers surveyed were also asked to provide data on the number of dry, single, twin and triplet-bearing ewes, where triplet-bearing ewes were identified. 

The producers that had some experience in separating and differentially managing twin- and triplet-bearing ewes were asked to rank the primary causes of mortality of triplet-bearing ewes and lambs, as well as the key practices they had adopted to improve survival of triplet-bearing ewes and/or triplet-born lambs. The practices described by producers were distilled into six key themes: (i) ewe condition score at lambing; (ii) FOO during lambing; (iii) ewe mob size during lambing; (iv) shelter during lambing; (v) handling pre-lambing, and disturbance before and during lambing; and (vi) supplementary feeding in late pregnancy with grain and/or minerals. Producers then identified which of these six strategies were their first, second and third priorities for reducing the mortality of triplet-bearing ewes and/or improving the survival of their lambs. Where appropriate, producers also provided the targets they adopted for ewe condition score, FOO and mob size at lambing, including how they compared to targets for twin-bearing ewes. In the context of this paper, a ‘mob’ refers to a group of sheep managed together within the same paddock. Finally, the producers were asked to identify key research priorities to improve the survival of triplet-bearing ewes and their lambs. 

### 2.2. Workshops and Webinar

Five workshops involving 78 producers were conducted across Australia in 2018, including at Katanning, WA (*n* = 8), Struan, SA (*n* = 5), Hamilton, VIC (*n* = 37), Ballarat, VIC (*n* = 13) and Holbrook, NSW (*n* = 15). There was also a webinar conducted involving a further 35 producers who could not attend the workshops. The workshops and webinar started by introducing the purpose of the project and a summary of the literature review on the survival of triplet-bearing ewes and their lambs [11]. A summary of the benchmark surveys was then presented regarding the proportion of triplet-bearing ewes in flocks across Australia, the mortality rates of both ewes and lambs and management practices being adopted for triplet-bearing ewes. Producers were then asked to respond using a Likert scale [15] regarding the relative importance or need for further research on 14 different management options identified from the literature review [11] and benchmarking surveys, and to individually rank their top priority for further research. These research priorities presented for ranking included: (i) ewe condition score at lambing; (ii) FOO during lambing; (iii) proportion of legume in the pasture on offer during lambing; (iv) use of alternative forages for lambing; (v) mob size during lambing; (vi) stocking rate during lambing; (vii) methods for supplementary feeding with grain; (viii) mineral supplementation; (ix) supplementation with specific nutrients such as vitamins and amino acids; (x) shelter options; (xi) intensive monitoring during lambing; (xii) mid-pregnancy shearing (demonstrated to increase lamb birthweight); (xiii) lamb fostering systems; and (xiv) quantification of foetal mortalities between pregnancy scanning and birth.

### 2.3. Statistical Analysis

All data were analysed using GENSTAT (Edition 22 [16]). Overall reproductive rate, marking rate, start of mating, length of mating and lamb survival were analysed using the method of Restricted Maximum Likelihood with ewe breed (Merino, non-Merino and mixed) and management system (‘separated’ or ‘combined’) at the farm level and flock level (nested within farm level) fitted as fixed effects, while year, state (nested within year), farm (nested within state) and flock (nested within farm) were fitted as random effects. 

Where farms identified and separated twin- and triplet-bearing ewes, ewe mortality and lamb survival for each birth type were analysed by the method of Restricted Maximum Likelihood with ewe breed at the flock level fitted as a fixed effect while year, state (nested within year), farm (nested within state) and flock (nested within farm) were fitted as random effects. Pearson correlation was used to measure the association between any two of the various parameters. The influence of the key management practice adopted by survey participants to reduce mortality of triplet-bearing and/or improve survival of their lambs on actual ewe mortality and lamb survival were analysed by the method of Restricted Maximum Likelihood with the main priority fitted as a fixed effect while year, state (nested within year) and farm were fitted as random effects.

## 3. Results

### 3.1. Survey Participants

Producers that completed surveys managed approximately 352,000 ewes, including 153,000 Merino ewes and 199,000 non-Merino ewes. Farm and flock demographic data for survey participants are shown in Table 1. On average, the farms managed by survey participants in NSW and WA were larger and a greater proportion of their farm was allocated to crops than the farms in VIC and SA. All farms in NSW and WA except one were mixed farms with both sheep and crops, whereas more than 50% of the farms in SA and VIC were specialist sheep producers with no crop. Participants with Merino ewes only had larger farms than those with non-Merino ewes only, and Merino ewes were more common on larger mixed farms with sheep and crops. Farms with both ewe breeds had, on average, fewer Merino than non-Merino ewes (1774 vs. 2173). One-third of the producers surveyed always managed multiple-bearing ewes together, and the key reasons given for not identifying triplet-bearing ewes were insufficient numbers of triplet-bearing ewes (68%), lack of capability of pregnancy scanning contractors (16%) and too many different mobs of ewes to manage (16%). There were no major differences in the total farm area, proportion of farm cropped or the total number of ewes between farms that had separated triplet-bearing from twin-bearing or always managed multiple-bearing ewes together.

At the farm level, participants with non-Merino ewes achieved a higher overall reproductive rate, lamb marking rate and lamb survival than those with Merino ewes only or a combination of both ewe breeds (Table 2). These estimates of reproductive rate at the farm level will be underestimated and lamb survival overestimated as the data includes combined flocks where triplet-bearing ewes were not identified, but the estimated means are adjusted for management system. On average, the start of the mating period was more than 2-weeks earlier for farms with both ewe breeds compared to farms with non-Merino ewes only, and the lamb marking rate achieved by farms with both breeds was intermediate between those with Merino or non-Merino ewes only. 

At the flock level, ewes were mated 17 to 22 days earlier but for 9 days longer on farms that always mixed multiple-bearing ewes together than ewes on farms that identified and separated at least some triplet-bearing ewes from twin-bearing ewes in either 2017 or 2018. There were no significant differences in flock reproductive rate, lamb marking rate or lamb survival between farms that always mixed multiple-bearing ewes together versus those who had identified and separated some triplet-bearing ewes from twin-bearing ewes in either 2017 or 2018. For farms that had separated some triplet-bearing ewes from twin-bearing ewes, flocks that were separated had a higher reproductive rate and marking rate, but there was no difference in reported lamb survival. Like above, this comparison needs to be treated with caution because the reproductive rate is underestimated, and lamb survival is overestimated in flocks where multiple-bearing ewes are combined. There were no significant interactions between ewe breed by ewe management system either at a farm level or flock within the farm level for overall reproductive rate, lamb marking rate or lamb survival. The raw means for Merino and non-Merino ewe flocks that identified and separated triplet-bearing ewes were 150.2% and 172.4% for reproductive rate, 112.8% and 137.4% for lamb marking rate and 75.1% and 79.9% for lamb survival.

### 3.2. Proportion of Triplet-Bearing Ewes

**Figure 2:**
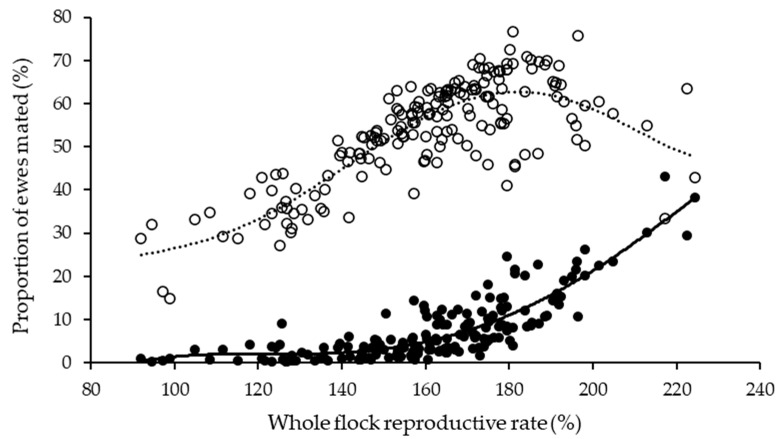
The proportion of twin-bearing (open circles) and triplet-bearing (solid circles) ewes relative to flock reproductive rate from survey data collected across the 2017 and 2018 breeding sesons in Australia. Raw data represents 145,000 Merino and non-Merino ewes.

The average reproductive rate was 163%, and 5.9% of ewes mated were identified as carrying triplets across all ewes managed by survey participants that provided the percentage of dry, single-, twin- and triplet-bearing ewes. The proportion of triplet-bearing ewes was significantly higher for non-Merino than Merino ewes (6.6% vs. 2.9%; *p* < 0.05), but at a given reproductive rate, the proportion of twin- and triplet-bearing ewes appeared to be similar for Merino and non-Merino ewes. The proportion of twin-bearing ewes increased to a maximum of around 60–65% at a corresponding reproductive rate of about 180%, and then started to decline as higher-order multiples increased (Figure 2). On average, the proportion of triplet-bearing ewes corresponding with reproductive rates of 140%, 160%, 180% and 200% were 2.2%, 5.7%, 11.4% and 21%, respectively. 

### 3.3. Survival of Triplet-Bearing Ewes and Their Lambs

Where triplet-bearing ewes were identified and managed separately from twin-bearing ewes, the average mortality of triplet-bearing ewes was 6.4% and ranged from 0% to 27% between individual flocks (10th percentile = 1.8% and 90th percentile = 14.5%). By contrast, the mortality of twin-bearing ewes was 3.3% and ranged from 0.5% to 17% (10th percentile = 1.2% and 90th percentile = 5.0%), and the mortality of single-bearing ewes was 1.6% and ranged from 0% to 8% (10th percentile = 0.5% and 90th percentile = 3.0%). There were no differences in the average mortality of single-, twin- or triplet-bearing ewes between ewe breeds (Table 3). Of the survey participants that reported the main causes of mortality of triplet-bearing ewes, 61% indicated pregnancy toxaemia, 55% indicated ewes being too heavy and 53% indicated dystocia. The reported causes of death were similar for triplet- and twin-bearing ewes, with the exception that only 24% of producers indicated that excessive liveweight was a major cause of death for twin-bearing ewes. 

The overall survival of triplet-born lambs was 59% and ranged from 34% to 79% between individual flocks (10th percentile = 45.3% and 90th percentile = 70.3%). By contrast, the average survival of twin-born lambs was 80% and ranged from 59% to 93% (10th percentile = 71.0% and 90th percentile = 88.6%), and the average survival of single-born lambs was 92% and ranged between 70% and 100% (10th percentile = 86.2% and 90th percentile = 96.8%). On average, the survival of lambs from non-Merino ewes tended to be higher for singles (*p* = 0.06) and was significantly higher for twins and triplets compared to their Merino counterparts (Table 3). Of the survey participants that reported the main causes of death for triplet-born lambs, 90% indicated mismothering, 68% low birthweight and 60% exposure to adverse weather conditions and hyperthermia. Fewer farmers reported that low birthweight was a significant cause of death for twin-born lambs (41%), but other differences in perceived causes of death between triplets and twins, including those likely to be related to birthweight, were minimal.

**Table 3:** Average survival of single, twin- and triplet-born lambs to marking and mortality of single, twin- and triplet-bearing ewes from pregnancy scanning to marking for Merino and non-Merino flocks where triplet-bearing ewes were managed separately from twin-bearing ewes. The data were derived from 105 flocks from 64 survey participants. Data for ewe mortality were angular transformed, and back-transformed values are presented.

	Lamb Survival (%)			Ewe Mortality (%)		
	Single	Twin	Triplet	Single	Twin	Triplet
Merino	89.9 ^a^	75.5 ^a^	52.9 ^a^	1.3 ^a^	2.5 ^a^	6.7 ^a^
Non-Merino	92.2 ^a^	81.4 ^b^	60.1 ^b^	1.5 ^a^	3.1 ^a^	4.9 ^a^
*p*-value	n.s.	<0.01	<0.01	n.s.	n.s	n.s.

Values within columns with different superscripts denote differences between ewe breeds (*p* < 0.05).

Farm area, proportion of farm cropped or the total number of breeding ewes had no significant effect on ewe mortality or lamb survival, regardless of litter size. Likewise, correlations between time of mating or length of the mating period and overall reproductive rate, marking rate and lamb survival or mortality of single, twins and triplets bearing ewes and their lambs, were generally not significant or very weak (Table 4). As expected, across all flocks, overall lamb marking rate was significantly correlated with both reproductive rate and lamb survival, especially survival of twin-born lambs. Furthermore, lamb survival was logically negatively correlated with ewe mortality for each litter size, especially for triplets.

Ewe mortality and lamb survival benchmarks for single-, twin- and triplet-bearing ewes and their lambs to achieve varying levels of overall lamb survival were generated by categorising the data and are summarised in Table 5. Survey participants that achieved less than 3.6% mortality of triplet-bearing ewes and 70% survival of triplet-born lambs achieved lamb survival rates across the whole flock of 90% or more.

### 3.4. Management Practices Adopted to Improve Survival of Triplet-Bearing Ewes and Their Lambs

About one-third of participants reported that the management of condition score of triplet-bearing ewes at lambing was their highest priority (Table 6). In addition, 80% of these producers indicated the primary reason for managing ewe condition score at lambing was to reduce ewe mortality, and almost 50% of these producers, who almost exclusively managed non-Merino ewes, indicated they tried to prevent ewes from getting over-fat. Mob size and shelter at lambing were the second and fourth highest priorities and, in all cases, smaller mobs and increased access to shelter were adopted to improve lamb survival rather than reduce ewe mortality. Management of FOO at lambing was the third highest priority, mostly to improve survival of lambs (46%) or to both improve survival of lambs and reduce mortality of ewes (39%). Reducing ewe handling pre-lambing and disturbance during lambing was seldom the highest priority, but about 30% of producers still included these strategies in their top three management priorities, primarily to reduce ewe mortality and improve lamb survival, respectively. The final strategies adopted to reduce both mortality of triplet-bearing ewes and improve survival of their lambs were supplementary feeding with grain and/or supplementation with minerals during pregnancy.

There were no significant differences in mortality of triplet-bearing ewes or survival of their lambs between producers that prioritised the adoption of certain management practices despite varying rates of adoption of these management practices (Table 6). Furthermore, there was substantial variation between participants in their targets for ewe condition score, mob size and FOO at lambing, and there were no significant correlations between actual condition score, mob size or FOO targets for triplets and mortality of triplet-bearing ewes or survival of triplet-born lambs.

### 3.5. Research Priorities to Improve Survival of Triplet-Bearing Ewes and Their Lambs

Survey participants that identified and separated at least some triplet-bearing ewes from twin-bearing ewes over the two-year period identified the need for further research on 14 different management options to reduce the mortality of triplet-bearing ewes and/or improve the survival of their lambs. The top four priorities identified for further research, which represented 73% of all research ideas, included targets for ewe condition score at lambing (31%), mob size during lambing (22%), FOO during lambing (11%) and mineral supplementation (9%). 

**Figure 3:**
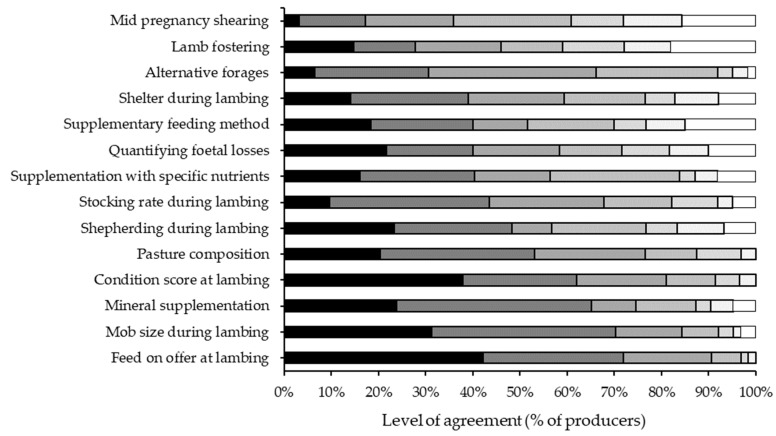
Percentage of respondents, on a seven-point scale ranging from strongly agree (on the left, black), agree, somewhat agree, neutral, somewhat disagree, disagree and strongly disagree (on the right, white), who believed further research was needed on different management options to reduce mortality of triplet-bearing ewes and/or improve the survival of their lambs. The respondents were sheep producers that attended workshops at sites across the sheep-producing regions of Australia or a webinar in 2019.

Similarly, the top four research priorities identified by producers during the workshops and a webinar were FOO during lambing, mob size during lambing, ewe condition score at lambing and mineral supplementation (Figure 3). Between 62 and 72% of producers indicated that further research was needed regarding each of these four management strategies. When producers identified a single management strategy as their highest research priority, these top four priorities again represented 78% of all responses: mob size during lambing (30%), ewe condition score at lambing (16%), FOO during lambing (16%) and mineral supplementation (16%).

## 4. Discussion

Consultation with sheep producers that had previously identified and differentially managed at least some triplet-bearing ewes indicated that reducing the mortality of triplet-bearing ewes was a high priority. The average mortality of triplet-bearing ewes from the benchmarking surveys was reported to be 6.4%, which was double that for twin-bearing ewes and four times that for single-bearing ewes. To our knowledge, this is the first study to quantify the mortality of triplet-bearing ewes on commercial farms across southern Australia. Kleemann et al. [12] reported an average mortality of 12% for triplet-bearing ewes across three years at a single research site, which is within the range for flocks in our study. As expected, the mortality of triplet-bearing ewes was strongly correlated with the survival of triplet-born lambs, whereas the correlation was weaker for twin- and single-bearing ewes and lambs. Indeed, the 3.1% higher mortality of triplet- compared to twin-bearing ewes was a key reason for the relatively small differences in overall lamb marking rates reported between triplet- and twin-bearing ewes (176 vs. 160%). The high average mortality of triplet-bearing ewes, together with the high frequency of farms with mortality rates greater than 10%, represents a significant loss of production for individual farms and an animal welfare risk for the sheep industry. Conversely, the 10th percentile for mortality of triplet-bearing ewes was only 1.8%, which indicates there is significant scope to reduce ewe mortality rates if the adoption of pregnancy scanning to identify triple-bearing ewes can be increased, and the components of best-practice management for these ewes can be identified and adopted. 

The average survival of triplet-born lambs from the benchmark surveys was reported to be 59%, which was 22% and 33% lower than their twin- and single-born counterparts. The survival of triplet-born lambs was considerably lower than the 68% survival reported across 29 research studies, mostly based in New Zealand, which could reflect, in part, that the data in our study were collected from commercial farms, whereas most of the data reported in Kenyon et al. [11] were from smaller-scale experiments and research farms. It is known that the survival of multiple-born lambs is lower at a commercial paddock scale compared to an experimental plot scale [17,18] or in larger mobs [6,7,8]. Furthermore, most of the studies reported by Kenyon et al. [11] utilised non-Merino ewes, and it was evident both in our data and Paganoni et al. [19] that the survival of both twin- and triplet-born Merino lambs was 5–10% lower than their counterparts from non-Merino ewes. The survival of triplet-born lambs varied from 35% to 79% between flocks, which is similar to that reported by Kenyon et al. [11]. Like ewe mortality, the 90th percentile for the survival of triplet-born lambs demonstrates the scope for improvement, particularly on some farms. Collectively, a survival rate of 70 to 75% for triplet-born lambs would seem to be an achievable target for extensive production systems in Australia where ewes lamb outdoors with minimal supervision. This is especially the case if the knowledge gaps identified by producers in this study can be addressed by further research and used to develop practical management guidelines for triplet-bearing ewes.

One-third of producers surveyed did not identify triplet-bearing ewes, and approximately two-thirds of these producers indicated the main reason for their decision was an insufficient number of triplet-bearing ewes. On average, each of these producers managed nearly 4000 breeding ewes and had they identified triplet-bearing ewes, their actual reproductive rate was likely to be around 153% rather than 148% based on scanning for multiples only. Therefore, it is likely thatapproximately 100 triplet-bearing ewes were mixed with the twin-bearing ewes in these flocks. As only 3% of all ewe flocks in Australia are scanned for triplet-bearing ewes [9], further work is clearly needed to understand the value proposition for producers to separate triplet- from twin-bearing ewes based on the numbers of ewes mated, current reproductive rates, farm characteristics and management capability. The value proposition will also be influenced significantly by the overall increases in lamb survival and weaning rate that can be achieved from separating triplet- from twin-bearing ewes compared to running all multiple-bearing ewes together. Lamb survival is overestimated in flocks where twin- and triplet-bearing ewes are combined, and in the current study the actual survival in these flocks across all lambs born was likely to be around 78% rather than 80%. In any case, this was still similar to the survival rate achieved by flocks where triplet- and twin-bearing ewes were separated, albeit from an estimated 11% lower reproductive rate. However, it is possible that the lack of difference in survival between flocks that did or did not differentially manage twin- and triplet-bearing ewes is because their differential management may not be optimal. Further research is needed to identify best-practice guidelines for triplet-bearing ewes, including quantifying the potential improvements in lamb survival from separating twin- and triplet-bearing ewes. Benefits could be substantial, especially if management guidelines for triplet-bearing ewes can be developed by addressing the research gaps identified by producers in this study. The non-economic advantages of adopting best practice management guidelines for triplets also need to be considered, including the ethical and emotional impacts of fewer ewe and lamb deaths and satisfaction from achieving greater productivity and profitability. 

A mixed-method approach involving more than 200 sheep producers from across southern Australia was effective at establishing the research needs and priorities of producers to improve the survival of triplet-bearing ewes and their lambs. Our hypothesis was therefore supported. The top four priorities for further research identified by producers were to establish targets for ewe condition score, FOO, mob size at lambing and quantify the impacts of supplementation with minerals, regardless of the ewe breed managed. These top four priorities represented between 73 and 81% of all responses, despite variations in the consultative processes used to identify the research priorities. The key research priorities to improve the survival of triplet-bearing ewes and their lambs identified from producer consultation in this study align with the knowledge gaps identified by Kenyon et al. [11]. Priorities for further work will also be informed by knowledge from the current study of management practices currently adopted by farmers, their production levels and the potential effect of changing a management practice on the mortality of triplet-bearing ewes and their lambs. These priorities will also be weighted based on the ease with which a management change can be achieved within the farming systems and hence the scale of the opportunity to reduce the mortality of triplet-bearing ewes and their lambs.

Most producers indicated the primary reason for managing the condition score of triplet-bearing ewes at lambing was to reduce ewe mortality. On average, the target condition score at lambing for triplet-bearing ewes of 3.3 was similar to that for twin-bearing ewes, but the target varied from 2.8 to 3.5 between producers. To our knowledge, there has been no detailed experimental work relating condition score profile during pregnancy and at lambing to risks of ewe mortality on commercial farms. In contrast to best practice management guidelines for twin-bearing Merino and non-Merino ewes, which require increased feeding to achieve a higher condition score at lambing for most farms [13,20,21], none of the producers involved in the benchmark surveys in our study indicated that low condition score at lambing contributed to mortality of triplet-bearing ewes, whereas almost 50% indicated they tried to prevent ewes from getting over-fat. Concern over multiple-bearing ewes getting too fat appeared to be a bigger issue for producers with non-Merino ewes than Merino ewes and for triplet- than twin-bearing ewes. This was consistent with beliefs that pregnancy toxaemia and ewes being too heavy at lambing were the main causes of mortality of triplet-bearing ewes. It is well recognised that over-fat ewes, especially those with multiple foetuses, are at greater risk of pregnancy toxaemia due to the direct and indirect effects of excessive fat on feed intake [22,23]. Optimising the condition score of triplet-bearing ewes will also be influenced by the impacts of condition score on lamb survival, and low birthweight was perceived to be a more dominant cause of mortality of triplet- compared to twin-born lambs by producers in our study. Studies in New Zealand and Australia using non-Merino ewes have found variable effects of ewe condition score at lambing on survival of triplet lambs [24,25,26,27], but most of these studies were small-plot scale on research stations rather than commercial scale and, in many cases, involved a limited range in condition score. Manipulating ewe condition score at lambing has a greater influence on the survival of twin-born lambs from Merino ewes compared to non-Merino ewes [17,27] due in part to lower average birthweights of lambs from Merino ewes [19], which may indicate a greater positive response of improving condition score in triplet-bearing Merino ewes than for non-Merino ewes. There is a clear need to better define the impacts of condition score at lambing on the mortality of triple-bearing ewes and their lambs at the commercial scale, and it is expected that these responses may differ between ewe breeds. 

Producers considered FOO at lambing important to reduce both ewe mortality and especially to improve lamb survival. Whilst the three-fold range in target FOO levels from 800 to 2500 kg DM/ha would, in part, reflect different production environments and time of lambing in relation to seasonal pasture supply, the producers indicated there was a need to better define the FOO targets for optimal survival of triplet-bearing ewes and their lambs. Studies in New Zealand found that offering around 800 kg DM/ha from mid-pregnancy until birth reduced birthweights of triplet-born lambs compared to higher FOO levels [28], but other studies have indicated that triplet-bearing ewes could be offered a minimum of 800 kg DM/ha without adverse effects on lamb survival provided intake was not restricted during the 2 weeks before lambing [24,26]. Another study from New Zealand showed a negative effect of offering triplet-bearing ewes a minimum of 1600 kg DM/ha in late pregnancy compared to 900 kg DM/ha on survival of triplet-born lambs, irrespective of ewe conditions score at mid-pregnancy [25]. As summarised by Kenyon et al. [11], literature regarding the effects of ewe nutrition and FOO during late pregnancy and lambing are variable. However, in many cases, studies are limited by low numbers of lambs per treatment, and few have subjected ewes to levels of nutrition well below their theoretical demand, which can occur in environments across southern Australia, especially when lambing in autumn or early winter. There are currently no industry recommendations for the FOO requirements for triplet-bearing ewes during late pregnancy and lambing under commercial farming conditions in Australia. More detailed studies are also needed to better understand the requirement for supplementary feeding triplet-bearing ewes in late pregnancy and lambing, depending on pasture conditions, to reduce ewe mortality and improve lamb survival.

Sheep producers reported that reducing mob size at lambing was a key practice they had adopted to improve the survival of triplet-born lambs, yet this remained a priority for further research. The average mob size at lambing from the benchmark surveys was reported to be 52 triplet-bearing ewes, but it was apparent that producers had very different opinions of the optimum mob sizes, which varied from 10–150 triplet-bearing ewes. Bates et al. [29] recently reported that mob sizes at lambing varied from 30–200 for triplet-bearing ewes across a small sample of farms, mostly in NSW. Lockwood et al. [7] reported survey data collected from sheep producers in southeastern Australia, which indicated the survival of single- and twin-born lambs increased by 1.4% and 3.5% when mob size at lambing was reduced by 100 ewes. This was verified by experimental data, which found that reducing mob size at lambing by 100 twin-bearing ewes increased the survival of their lambs by 1.9 to 2.5%, regardless of breed and stocking rate at lambing [8]. The optimum mob size for twin-bearing ewes was typically less than half that for single-bearing ewes depending on several enterprise-specific factors [14]. There are currently no recommendations for the optimum mob size during lambing for triplet-bearing ewes [11]. Mismothering was perceived to be the main cause of death for triplet-born lambs in the current study, and the effects of mob size on lamb survival are likely to be driven by the risk of mismothering. The risk of mismothering is likely to be greater for triplet-born than twin- or single-born lambs, given more triplet lambs will be born per day for the same ewe mob size, triplet-born lambs and their dams have poorer behavioural traits than both singletons and twins [30,31,32], and triplet-bearing ewes have been observed to take longer to deliver their litter than twin- and single-bearing ewes [33]. More detailed studies to quantify the effects of mob size on lamb survival are needed to underpin economic modelling and determine the optimal mob size for triplet-bearing ewes for specific management settings.

Little is known regarding whether mineral supplementation of lambing ewes can reduce ewe and lamb mortality. Subclinical deficiencies of calcium and magnesium are common in lambing ewes in Australia due to imbalances in pasture grazed by the ewes [34]. Mineral imbalances in vegetative cereal crops also present a risk of low calcium status in ewes grazing these crops in late pregnancy [35]. Subclinical deficiencies in calcium and magnesium may increase the risk of dystocia and related issues, including hypothermia in lambs and poor ewe-lamb behaviour [34]. Providing lambing ewes with ad libitum access to mineral supplements containing magnesium, sodium and calcium has been reported to reduce the risk of ewe mortality when grazing cereal crops [36]. However, the impact of subclinical mineral deficiencies on lamb mortality and the benefits of mineral supplementation remains unclear. It could be assumed that the benefits of mineral supplementation would be greater for triplet-bearing ewes and their lambs compared to their single- or twin-counterparts, given their higher metabolic demands during pregnancy and lactation.

The sheep producers consulted in this study represented a biased sample, so caution is needed in extrapolating some of the findings across the national sheep flock, especially those relating to levels of reproductive performance. Producers must have utilised pregnancy scanning for litter size to be eligible for inclusion in the benchmarking surveys, and this only represents about 35% of Australian sheep producers [9]. Furthermore, many producers known to have experience with differential management of triplet-bearing ewes were deliberately targeted. The average reproductive rates for farms that identified triplet-bearing ewes were 150% for Merinos and 172% for non-Merinos, which were significantly higher than the industry average reproductive rates of 122% and 147%, respectively [9]. The average proportion of triplet-bearing ewes for these farms was over 6%, which is likely to be about double that present across the national flock, but nevertheless, it is still likely that 1 to 1.5 million ewes conceive triplets annually across Australia given that the flock size is approaching 45 million breeding ewes. As all of the producers surveyed had adopted pregnancy scanning for multiples, it is likely that they had also adopted other management strategies to improve reproductive performance compared to the broader population of sheep producers [5,6]. Despite higher reproductive rates and, therefore, more multiple-born lambs, the average lamb survival rates for farms in this study were 75% for Merinos and 80% for non-Merinos, which were significantly higher than the industry average lamb survival rates of 69% and 71%, respectively [9]. Mortality rates of triplet-bearing ewes and their lambs across the sheep industry in Australia are likely to be higher than reported in our study due to survey recall bias associated with self-reported retrospective surveys. Munoz et al. [37] recently reported that a cohort of 32 farmers from across Victoria in southeastern Australia reported their annual ewe mortality was 2.7% compared to 4.7% based on changes in actual sheep numbers over a calendar year. Furthermore, our data suggest that mortality rates of triplet-born lambs are likely to be higher in flocks where triplet-bearing ewes are mixed with twin-bearing ewes, especially when none of the ewes are pregnancy scanned. It is clear that changes in profitability from improving the survival of triplet-bearing ewes and their lambs will be relatively small for most farms where triplet-bearing ewes represent less than 5% of the ewe flock. The increasing prevalence of triplet-bearing ewes, however, means that identifying and adopting best-practice management of triplet-bearing ewes and their lambs will be important for improved productivity and to ensure animal welfare is optimised to meet consumer demands for ethical product.

## 5. Conclusions

Surveys completed by 64 producers across Australia who separated triplet-bearing ewes from twin-bearing ewes found that 5.9% of all ewes mated were carrying triplets. The average mortality of triplet-bearing ewes of 6.4% did not differ between ewe breeds, whereas survival of triplet-born lambs was 60.1% for those from non-Merino ewes compared to 52.9% for Merino ewes. The key strategies adopted to reduce the mortality of triplet-bearing ewes and their lambs were the management of condition score at lambing, FOO and mob size during lambing, and use of shelter. Overwhelmingly, the highest priorities for further research identified by producers were ewe condition score, mob size, FOO at lambing and mineral supplementation. 

## Figures and Tables

**Table 1:** Number of farms, average size of farms, proportion of the farms cropped and total number of ewes mated for survey participants across Australia in 2017 and 2018. The farm-level benchmarking data are presented for different states, ewe breed types on each farm (Merino, non-Merino or both) and the management system utilised for triplet-bearing ewes (‘Separated’ or ‘Combined’ with twin-bearing ewes). The ewe management system for a farm was classified as ‘Separated’ if the triple-bearing ewes for at least one ewe flock were identified and separated from twin-bearing ewes in either 2017 or 2018.

	Number of Farms	Farm Area (Total Ha)	Crop Area (% Farm)	Ewes Mated (Head)
State				
New South Wales	18	2234	41.6	3226
Victoria	43	1652	12.4	3631
South Australia	15	1373	6.6	2924
Western Australia	19	3072	36.8	4974
Farm ewe breed				
Merino	25	3178	34.2	4713
Non-Merino	50	1286	14.7	3116
Merino and non-Merino	20	2324	23.0	3947
Farm management system for triplet-bearing ewes				
Separated	64	1891	20.4	3608
Combined	31	2210	23.6	3925

**Table 2:** Average date at start of mating period, length of mating period, reproductive rate (foetuses per 100 ewes mated; 245 flocks), lamb marking rate (lambs marked per 100 ewes mated; 240 flocks) and lamb survival (lambs marked per 100 foetuses scanned; 227 flocks) for survey participants in 2017 and 2018. The data are presented for farms with different ewe breeds (Merino, non-Merino or both), and for flocks where multiple bearing ewes were always combined together (‘Combined/Combined’), flocks where multiple bearing ewes were combined from farms that separated some triplet-bearing ewes from twin-bearing ewes in either 2017 or 2018 (‘Combined/Separated’), and flocks were twin- and triplet-bearing ewes were always separated (‘Separated/Separated’).

	Start of Mating (Date)	Length of Mating (Days)	Reproductive Rate (%)	Lamb Marking Rate (%)	Lamb Survival (%)
Farm ewe breed					
Merino	24 January ^ab^	44 ^a^	144.5 ^a^	112.7 ^a^	78.2 ^a^
Non-Merino	5 February ^a^	46 ^a^	165.7 ^b^	136.5 ^b^	82.0 ^b^
Merino and non-Merino	16 January ^b^	44 ^a^	148.1 ^a^	118.3 ^c^	77.9 ^a^
*p*-value	<0.05	n.s.	<0.001	<0.001	<0.01
Ewe flock management system					
Combined/Combined	16 January ^a^	49 ^a^	146.3 ^a^	121.2 ^a^	80.8 ^a^
Combined/Separated	7 February ^b^	40 ^b^	148.8 ^a^	117.1 ^a^	79.0 ^ab^
Separated/Separated	2 February ^b^	40 ^b^	164.4 ^b^	127.1 ^b^	77.5 ^b^
*p*-value	<0.01	<0.001	<0.001	n.s.	<0.05

Values within columns with different superscripts (a, b, c) denote differences between farms with different ewe breeds or ewe flock management systems (*p* < 0.05).

**Table 4:** Correlations between start date and length of the mating period, overall flock reproductive rate, marking rate and lamb survival, mortality of single-, twin- and triplet-bearing ewes and survival of their lambs to marking. The data were derived from 105 flocks of Merino and non-Merino ewes from 64 survey participants, where triplet-bearing ewes were managed separately from twin-bearing ewes.

	Start of Mating (Date)	Length of Mating (Days)	Flock Reproductive Rate (%)	Flock Lamb Marking Rate (%)	Flock Lamb Survival (%)	Single Ewe Mortality (%)	Single Lamb Survival (%)	Twin Ewe Mortality (%)	Twin Lamb Survival (%)	Triplet Ewe Mortality (%)
Length of mating	0.10									
Flock reproductive rate	0.20	0.21								
Flock lamb marking rate	0.29 **	−0.01	0.73 ***							
Flock lamb survival	0.17	−0.28 *	−0.15	0.56 ***						
Single ewe mortality	−0.10	−0.09	0.07	−0.17	−0.34 **					
Single lamb survival	0.18	0.14	0.41 ***	0.55 ***	0.32 **	−0.30 **				
Twin ewe mortality	−0.09	0.07	0.14	−0.19	−0.42 ***	0.71 ***	−0.12			
Twin lamb survival	0.13	−0.25 *	0.30 **	0.71 ***	0.59 ***	−0.31 **	0.41 ***	−0.31 **		
Triplet ewe mortality	−0.15	0.17	0.06	−0.24 *	−0.44 ***	0.32 **	−0.01	0.48 ***	−0.39 ***	
Triplet lamb survival	0.28 **	−0.17	0.09	0.47 ***	0.67 ***	0.30 **	0.26 *	−0.38 ***	0.56 ***	−0.63 ***

* *p* < 0.05; ** *p* < 0.01; *** *p* < 0.001.

**Table 5:** Lamb survival and ewe mortality benchmarks corresponding to varying rates of lamb survival across whole flocks. The data were derived from 105 flocks from 64 survey participants that identified and differentially managed twin- and triplet-bearing ewes between pregnancy scanning and lamb marking in 2017 and 2018. Data include Merino and non-Merino ewe flocks.

Overall Lamb Survival (%)	Lamb Survival (%)			Ewe Mortality (%)		
	Single	Twin	Triplet	Single	Twin	Triplet
70 (67.5–72.5)	88.5	73.5	51.9	2.0	4.0	7.5
80 (77.5–82.5)	92.9	81.1	59.0	1.3	3.0	5.2
90 (87.5–92.5)	92.8	87.8	69.6	1.1	2.0	3.6

**Table 6:** The proportion of producers that identified different practices as their first, second and third priorities to reduce mortality of triplet-bearing ewes and/or improve survival of their lambs, their recommendations (average and range) for condition score, mob size and feed-on-offer (kg dry matter/ha) at lambing for twin- and triplet-bearing ewes, and the average mortality of triplet-bearing ewes and survival of their lambs for producers that identified the management practice as their first priority. Data were collected from 64 participants of the benchmarking surveys conducted in 2017 and 2018 for producers that had pregnancy scanned to identify triplet-bearing ewes and managed them separately from twin-bearing ewes.

Management Practice	Respondents (%)				Recommendations		Triplet Ewe Mortality (%)	Triplet Lamb Survival (%)
	First Priority	Second Priority	Third Priority	Total	Triplet-Bearing Ewes	Twin-Bearing Ewes		
Condition score at lambing	34	11	9	51	3.3 (2.8–3.5)	3.2 (2.9–3.8)	5.1 ^a^	61.7 ^a^
Mob size during lambing	23	30	21	64	52 (10–150)	134 (50–250)	6.1 ^a^	58.7 ^a^
Feed on offer at lambing	20	28	14	54	1710 (800–2500)	1530 (800–2200)	4.6 ^a^	58.5 ^a^
Shelter during lambing	16	19	7	44	-	-	6.5 ^a^	58.8 ^a^
Ewe handling and monitoring	3	6	33	31	-	-	-	-
Supplementary feeding	2	7	7	14	-	-	-	-

^a^ Values within columns are not statistically significant (*p* > 0.05).

## Data Availability

The datasets generated and/or analysed during the current study are not publicly available but are available from the corresponding author on reasonable request, pending permission from the funding body (Meat and Livestock Australia) and Murdoch University.
